# Demographics, clinical presentation and risk factors of ocular surface squamous neoplasia at a tertiary hospital, South Africa

**DOI:** 10.1038/s41433-023-02565-1

**Published:** 2023-05-31

**Authors:** Roland Hӧllhumer, Pamela Michelow, Susan Williams

**Affiliations:** 1 Department of Neurosciences, Division of Ophthalmology, University of the Witwatersrand, Johannesburg, South Africa; 2 The Cornea Foundation, Johannesburg, South Africa; 3 Cytology Unit, National Health Laboratory Service and Department of Anatomical Pathology, Faculty of Health Sciences, University of the Witwatersrand, Johannesburg, South Africa

**Keywords:** Eye manifestations, Risk factors, Conjunctival diseases, Eye cancer

## Abstract

**Aims:**

The aim of this study is to describe the demographic, presenting features and associated risk factors of ocular surface squamous neoplasia (OSSN) at a tertiary eye hospital in Johannesburg, South Africa.

**Methods:**

An interventional prospective study of patients presenting with conjunctival masses was conducted. An electronic questionnaire was completed to document demographic data, presenting history, and associated risk factors. A slit lamp examination and photos were used to document and describe the clinical features at presentation. Cases (OSSN) and controls (benign lesions) were determined by histology.

**Results:**

There were 130 cases and 45 controls. Median age was 44 years (IQR: 35–51) with an equal gender distribution in cases. The prevalence of HIV in cases was 74% and was strongly associated with OSSN (*p* < 0.001). Vascularisation, leukoplakia and pigmentation were clinical features that distinguished cases from controls. A fibrovascular morphology was strongly associated with a benign histology (*p* < 0.001), whereas leukoplakic and gelatinous morphologies were associated with OSSN. Conjunctival intra-epithelial neoplasia made up 82% of cases.

**Conclusion:**

Our study describes a sample of OSSN that is young and has no gender predisposition. The majority of cases presented with CIN lesions, rather than SCC reported in other African countries. HIV was the most significant risk factor in this study population.

## Introduction

Ocular surface squamous neoplasia (OSSN) is an umbrella term for a group of conjunctival tumours that include conjunctival intra-epithelial neoplasia (CIN) and squamous cell carcinoma (SCC) [1]. It is the most common ocular surface tumour with incidence rates ranging from 0.03–1.9 per 100,000 persons/year in high-income countries (HIC) and 1.6–3.4 per 100,000 persons/year in sub- Saharan Africa [1–3]. OSSN presents in two main demographic groups, older white males in HIC and younger black females in low- and middle-income countries (LMIC) [3]. The mean age of presentation in HIC is 56 years and in LMIC this mean is in the 30s [1, 3]. Sub-Saharan Africa typically falls into the latter group where human immunodeficiency virus (HIV) and human papilloma virus (HPV) are thought to play a larger aetiological role [3].

**Fig. 1:**
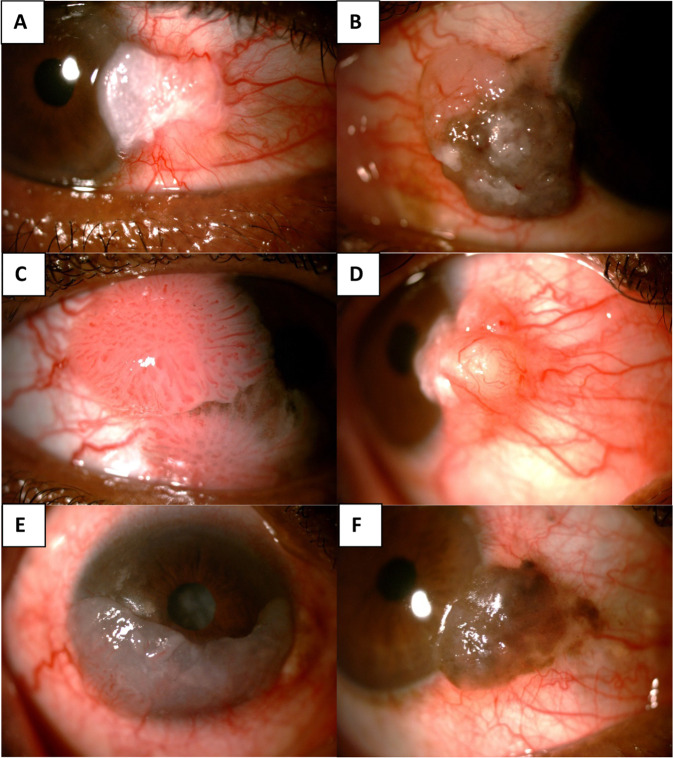
Morphological subtypes of OSSN. **A** Leukoplakic, **B** gelatinous with some leukoplakia and pigmentation, **C** papilliform, **D** nodular, **E** diffuse, **F** pigmented OSSN.

OSSN presents with non-specific symptoms such as redness and ocular irritation. If the lesion is large it may cause visual impairment by obstructing the visual axis or inducing astigmatism [1, 4]. OSSN is suspected clinically in patients with conjunctival masses that are raised, increasing in size and have feeder vessels [1]. Morphologically they are classified into placoid, nodular and diffuse lesions (Fig. 1) [2, 5]. Placoid lesions are further divided into leukoplakic, gelatinous and papilliform lesions [2, 5]. An OSSN lesion may exhibit more than one morphology. The gold standard for confirmation of the diagnosis and histological grading is a biopsy with histological evaluation [6].

The two leading risk factors that have been associated with OSSN are ultra-violet-B radiation (UVB) and HIV infection [7]. HPV has an uncertain association, with some studies showing a strong association and others showing no association [4]. Several other associations of uncertain significance have been described, and include smoking, exposure to petroleum products, vitamin A deficiency, hepatitis B and C infection, ocular injuries, chronic ocular surface inflammation, and immunodeficiency other than HIV [2, 8, 9].

There is a paucity of reported data on OSSN in South Africa. The aim of this paper is to describe the demographic, presenting features and associated risk factors at a tertiary eye hospital in Johannesburg, South Africa.

## Materials and methods

An interventional prospective case-control study of patients presenting with conjunctival masses at a tertiary eye hospital in Johannesburg, South Africa was conducted. South Africa is a middle-income country [10]. Ethics approval was granted by the Human Research Ethics committee of the University of the Witwatersrand (M190729) and the study adhered to the tenets of the declaration of Helsinki.

Patients that presented with conjunctival masses suspicious of OSSN or symptomatic conjunctival growths between December 2019 and February 2022 were considered for inclusion in the study. Conjunctival masses were considered to be suspicious of OSSN if they had morphology typical of OSSN or had feeder vessels, excess pigmentation or were increasing in size. Benign appearing masses were offered surgical excision if they caused astigmatism or were symptomatic despite medical management. Exclusion criteria included: age less than 18 years; pregnancy or breastfeeding; previous surgery or topical chemotherapy in the presenting eye; masses greater than 15 mm in basal diameter or invading adjacent structures (other than cornea or sclera); neurological conditions that prevented study investigations; heritable conditions that predispose to OSSN (xeroderma pigmentosum, oculocutaneous albinism); and primary acquired melanosis.

Patients were enrolled after informed consent was taken. An electronic questionnaire was completed at enrolment to document demographic data, presenting history, and associated risk factors. The questionnaire was in English, with a translator present when needed. Associated risk factors that were enquired on include: HIV and the use of ARVs; other systemic conditions or medications associated with immunosuppression; average time spent outdoors every day; use of tobacco products; previous ocular injury; previous or current chronic inflammatory ocular surface disease; and history of regular exposure to petroleum products.

A slit-lamp examination was performed to identify the clinical features, dimensions, and morphology of the mass. The dimensions were recorded as the largest two perpendicular dimensions, with surface area calculated from these. The limbal clock hour involvement was recorded. In the absence of ultrasound biomicroscopy (UBM), the mass thickness was compared to the lid margin, where the lid was assumed to be 1.5 mm thick. An anterior segment photo was taken to document clinical features and pigmentation. Pigmentation of the masses was recorded as a percentage pigmentation of the surface of the mass. Pigmentation was described as mild if 0–33% of the mass was pigmented, moderate when this was 34–66% and severe when 67–100%. In patients with poor clinical visualisation of the margins, methylene blue stain was used to delineate the margins and calculate the area of pigmentation. Routine blood investigations included: voluntary HIV testing with CD4 count and viral load if reactive. Additionally, 100 participants (34 controls and 66 cases) had hepatitis-B serology, hepatitis-C serology, vitamin A levels and C-reactive protein (CRP) done. The CRP was done to assess if HIV patients with low vitamin-A levels were in the acute phase response that would result in a false low vitamin-A level. If any abnormality was detected on these investigations, the patient was referred to internal medicine or infectious disease for further management.

All patients had a biopsy to determine the diagnosis. Histology showing CIN, squamous cell carcinoma in situ (CiS) or SCC made up the cases whereas benign lesions on histology made up the controls. Participants that had bilateral surgery during the course of the study only had the first eye included for statistical analysis.

Data analysis was performed using STATA (StataCorp LLC, Texas, USA), version 17.0. Descriptive statistics were used for patient characteristics, clinical features, and associated risk factors. Shapiro-Wilk test was used to test for normality. Categorical data were presented as numbers and percentages. Continuous data that do not show a normal distribution were summarised with medians and interquartile range. Wilcoxon rank sum test was used to compare continuous variables that do not have a normal distribution. The chi-square or Fisher’s exact test was used to compare categorical variables. Univariate logistic regression analysis was used to identify clinical findings and previously reported risk factors of OSSN. The results of the logistic regression analysis were presented with OR with 95% CI. Significance levels was set up at *p* < 0.05.

## Results

One hundred and seventy-five patients were recruited during the study period (Supplementary 1). There were 130 cases and 45 controls. Table 1 gives an overview of the demographics, symptoms, presenting features, clinical signs, histology and associated risk factors. The median age at presentation for cases and controls was 44 and 49 years (*p* = 0.02) with an almost equal distribution between males and females.

**Table 1:** Comparison of demographics, symptoms, presenting characteristics, signs, histology and associated risk factors between cases and controls.

	Cases (*n* = 130)	Controls (*n* = 45)	*p* value
Demographics			
Median age in years (IQR)	44 (35–51)	49 (40–56)	0.02
Sex (%)			0.53
Male	62 (48)	19 (42)	
Female	68 (52)	26 (58)	
Race (%)			0.30
Black African	127 (98)	45 (100)	
Mixed Race	3 (2)	0	
Symptoms			
Symptoms (%)			
Foreign body sensation	43 (33)	19 (42)	0.27
Pain	40 (31)	6 (13)	0.02
Reduced vision	14 (11)	13 (29)	0.004
Redness	19 (15)	12 (27)	0.07
Itching	30 (23)	10 (22)	0.91
Epiphora	12 (9)	2 (4)	0.53
Photophobia	8 (6)	3 (7)	0.82
Median duration of symptoms in months (IQR)	2 (1–7)	12 (4–48)	<0.001
Presentation			
Conjunctival mass noticed by patient (%)	108 (83)	42 (93)	0.09
Median delay from presentation to first healthcare facility to referral for ophthalmic care in days (IQR)	2 (1–6)	2 (1–18)	0.49
Signs			
Laterality (%)			
Right	61 (47)	25 (56)	0.32
Left	69 (53)	20 (44)	0.32
Mass location (%)			
Epicentre			0.23
Conjunctiva	58 (45)	20 (44)	
Limbus	72 (55)	24 (53)	
Cornea	0	1 (2)	
Quadrants (%)			
Nasal	106 (81)	40 (89)	0.25
Temporal	28 (22)	4 (9)	0.06
Superior	5 (4)	0	0.33
Inferior	11 (8)	1 (2)	0.30
Clinical features (%)			
Vascularised	119 (92)	35 (78)	0.01
Elevated	120 (92)	41 (91)	0.8
Leukoplakia	67 (52)	6 (13)	<0.001
Pigmented	71 (55)	11 (24)	<0.001
Mild (0–33%)	28 (22)	9 (20)	0.83
Moderate (34–66%)	16 (12)	7 (16)	0.58
Severe (67–100%)	19 (15)	4 (9)	0.33
Morphology (%)			
Fibrovascular	15 (12)	31 (69)	<0.001
Nodular	4 (3)	2 (4)	0.65
Diffuse	2 (2)	0	1.00
Placoid	110 (85)	12 (27)	<0.001
Leukoplakic	67 (61)	6 (50)	<0.001
Gelatinous	45 (41)	5 (42)	0.003
Papilliform	14 (13)	2 (17)	0.25
Median surface area, mm^2^ (IQR)	22 (9–33.4)	17.5 (12.25–22)	0.01
Median limbal clock hours involved (IQR)	2 (1–3)	2 (1–3)	0.68
Tumour thickness (%)			0.06
<1.5 mm	89 (68)	39 (87)	
=1.5 mm	16 (12)	3 (7)	
>1.5 mm	25 (19)	3 (7)	
Scleral fixation (%)	19 (15)	1 (2)	0.027
Histology			
Benign (%)		45	
Pterygium		43 (96)	
Papilloma		1 (2)	
Naevus		1 (2)	
OSSN (%)			
CIN 1	34 (26)		
CIN2	34 (26)		
CIN 3	39 (30)		
CiS	6 (5)		
SCC	17 (13)		
Clinically considered to be benign (%)	15 (12)	31 (69)	
Clinically considered to be OSSN (%)	115 (88)	14 (31)	
Associated risks			
Declined HIV testing	4 (3)	3 (7)	0.38
HIV positive (%)	93 (74) (*n* = 126)	14 (32) (*n* = 42)	<0.001
Known at presentation	65 (70)	11 (79)	0.47
Newly diagnosed	28 (30)	3 (21)	0.96
Median CD4 at presentation, cells/ul (IQR)	202 (120–415)	595 (270–722)	0.003
Median VL at presentation, log copies/ml (IQR)	2.86 (1–4.76)	1 (1–2.02)	0.004
On ARVs	56 (86)	11 (100)	0.19
Median duration of ARV use in months (IQR)	29.5 (11–95)	93 (55–131)	0.02
Tobacco use (%)	26 (20)	11 (24)	0.53
Cigarettes	19 (73)	9 (82)	
Snuff	6 (23)	2 (18)	
Other	2 (8)	0	
Median hours of sun exposure (IQR)	3 (2–8)	3 (1–6)	0.31
Ocular trauma (%)	5 (4)	1 (2)	1.00
Chronic ocular surface inflammatory disease (%)	3 (2)	0	0.57
Petroleum exposure (%)	1 (1)	0	1.00
Median vitamin A levels^a^ (IQR)	1.6 (1.38–2.1) (*n* = 66)	1.67 (1.45–2.38) (*n* = 34)	0.47
Hepatitis B (%)	8 (12) (*n* = 66)	1 (3) (*n* *=* −34)	0.16
Hepatitis C (%)	0 (*n* = 66)	0 (*n* = −34)	

Vitamin A, hepatitis B and hepatitis C were done for 100 participants (66 cases and 34 controls).

*IQR* interquartile range, *CIN* conjunctival intra-epithelial neoplasia, *CiS* squamous cell carcinoma in situ, *SCC* squamous cell carcinoma, *HIV* human immune-deficiency virus, *VL* viral load, *ARV* anti-retro viral.

^a^All vitamin A levels below normal had CRP reviewed to exclude a false low level due to the acute phase of an infection (none had this). Normal range for vitamin A is 1.05–2.8 umol/l.

Symptoms were similar between the two groups (Table 1), except for pain and reduced visual acuity. Pain occurred more commonly in the cases (*p* = 0.02) whereas the controls reported poorer visual acuity (*p* = 0.004).

Vascularisation, leukoplakia and pigmentation were clinical features that distinguished OSSN from benign masses (Table 1). A fibrovascular morphology was strongly associated with a benign histology (*p* < 0.001), whereas leukoplakic and gelatinous morphologies were associated with OSSN. Scleral fixation of the mass on clinical assessment was associated with OSSN (*p* = 0.027). We found OSSN in 31% of cases that were clinically suspected to be pterygia and 12% of clinically suspected OSSN cases were found to be benign on histology.

HIV infection was strongly associated with OSSN (*p* < 0.001) (Table 1). A lower viral load, CD4 count, and shorter duration of ARV use was associated with OSSN (Table 1). Table 2 highlights the relationship between HIV, CD4 count, viral load and use of ARVs in cases and controls. There was a decreasing trend of CD4 count with more advanced histology of OSSN. There was no statistically significant difference in sun exposure between cases and controls (*p* = 0.31).

**Table 2:** Comparison of histology to HIV serology, CD4 count viral load and number of patients that have commenced ARVs.

Histology	HIV *n* (%)	Median CD4	Median viral load	ARVs
Benign (*n* = 47)	14 (30)	595	19	79%
CIN 1 (*n* = 36)	18 (50)	300	402	72%
CIN 2 (*n* = 35)	25 (71)	208	4587	60%
CIN 3 (*n* = 41)	35 (85)	153	342	57%
CiS (*n* = 6)	6 (100)	279	23,050	50%
SCC (*n* = 17)	14 (82)	185	168	57%

Table 3 documents a regression analysis that was performed to describe clinical features and risk factors associated with OSSN in our cohort.

**Table 3:** Regression analysis of clinical features and risk factors for OSSN.

Risk factor	Odds ratio	95% confidence interval	*p* value
Pain	2.9	1.1–7.4	0.03
Vascularised	3.1	1.2–7.9	0.02
Fibrovascular	0.1	0.0–0.1	<0.001
Leukoplakia	6.9	2.7–17.5	<0.001
Gelatinous	4.2	1.6–11.5	0.005
Pigmentation	3.7	1.7–8.0	0.001
Scleral fixation	7.5	1.0–58.0	0.05
HIV	6.4	3.0–13.7	<0.001
CD4 count	1.0	0.9–1.0	0.06
Viral load	2.1	1.1–4.0	0.02
Sun exposure	1.1	0.9–1.2	0.34

## Discussion

OSSN is the most common ocular surface tumour [3]. It has two major patterns of presentation, an older white male, and younger black female population [3]. The leading risks associated with OSSN are UVB exposure and HIV infection [4]. Our study describes the demographic pattern, clinical presentation and associated risks in an urban South African population.

The demographics of OSSN differ according to regions. The traditional description of OSSN demographics has been dichotomous with HICs (previously ‘developed’ countries) describing an older white male population where UVB exposure is the main risk factor and LMICs (previously ‘developing’ countries) describing a younger female population [3]. This difference has been ascribed to HIV which disproportionately affects LMICs and is a known risk factor for OSSN [3, 8]. Our study (from South Africa, a middle-income country) found OSSN in a young population with a median age of 44 years but no particular gender predilection. Table 4 summarises the age and gender associations with OSSN that have been reported, in the literature, from different regions and relates these to the gross national income (GNI) per capita and HIV prevalence of the country (and in the year) in which the research was conducted. As can be seen, it is necessary for us to update our classification of OSSN into low-, middle- and high-income countries. The low-income countries demonstrate the young, female demographic in OSSN prevalence, the high income the older male demographic, with middle-income countries falling between the two with an intermediate age and no gender predilection. Our study’s young population can be explained by the high HIV prevalence that likely resulted in an earlier age of presentation in South Africa (as with Botswana). In common with other middle-income countries our cohort did not show a gender predilection. Future studies could further investigate the relationship between income, risk factors and OSSN.

**Table 4:** Comparison of gross national income, HIV infection rates, demographics and histology in OSSN for studies conducted in low-, middle- and high-income countries.

Country	GNI per capita^a^	HIV prevalence	Mean age	Male: Female	Histology
Low-income countries					
Kenya (2015) [17]	$1300	2.6%	41 years	1:2	CIN 37% SCC 63%
Kenya (2016) [8]	$1460	2.6%	42 years	1:2.6	
Kenya (2017) [29]	$1510	2.6%	42 years	1:1.8	
India (2015) [13]	$1600	0.19%	40 years	1:0.4	CIN 35% SCC 65%
Nigeria (2021) [11, 20]	$2100	1.4%	48 years	1:1	CIN 16% SCC 84%
Nigeria (2010) [21]	$2150	4.6%	39 years	1:1.5	
India (2022) [14]	$2170^b^	0.19%	49 years	1:0.5	CIN 56% SCC 44%
Middle-income countries					
Botswana (2015) [22]	$6430	15.7%	38 years	1:1.4	CIN 43% SCC 57%
** South Africa (current study)**	**$6440**^**b**^	**13.7%**	**44 years**	**1:1.1**	**CIN 82%** **SCC 18%**
Thailand (2022) [15]	$7260^b^	0.69%	59 years	1:0.5	CIN 54% SCC 46%
Mexico (2022) [30]	$9380^b^	0.26%	66 years	1:0.8	CIN 64% SCC 36%
High-income countries					
New Zealand (2021) [31]	$45,340	0.06%	69 years	1:0.3	CIN 70% SCC 30%
USA (2012) [32]	$52,790	0.4%	71 years	1:0.5	CIN 84% SCC 16%
USA (2020) [27]	$64,140	0.4%	66 years	1:0.8	
USA (2021) [18]	$70,430	0.4%	71 years	1:0.25	

*GNI* gross national income, *HIV* human immunodeficiency virus, *CIN* conjunctival intra-epithelial neoplasia, *SCC* squamous cell carcinoma.

^a^GNI data from the The World Bank [10], HIV data from UNAIDS (data used from closest year) [12].

^b^GNI data from 2021 used, as 2022 data not yet available.

The bold row highlights data from the current study.

OSSN can be asymptomatic or present with symptoms ranging from redness, foreign body sensation, pain, epiphora and reduced vision [1, 11, 12]. Our cases reported a similar symptom profile between the cases and controls. Pain was however a distinguishing feature with more pain reported in cases. Reduced vision and redness were reported to a greater degree in the control group, but this was likely due to selection bias as the controls were only offered surgery if they had significant astigmatism or persistent symptoms despite medical therapy.

The epicentre of OSSN in our study was largely at the limbus and in the conjunctiva. This is in keeping with large studies and follows the pathophysiology of OSSN, which is thought to originate from the limbal stem cells [7, 13–16]. OSSN is also most commonly reported in the interpalpebral space, with the nasal quadrant mostly affected [14, 15]. This is hypothesised to be due to the amplification of UVB radiation on the nasal limbus [16]. Our results were in keeping with this, where the nasal quadrant was mostly affected followed by the temporal quadrant. On clinical examination there were several features that were significantly associated with OSSN. These include a mass with feeder vessels, leukoplakia and pigmentation. The large number of pigmented lesions in our study is likely due to our predominantly black African cohort where more pigmentation has been reported than in white patients [17]. Pigmentation in OSSN lesions has a significant regional variation and has been reported at 1% in a white cohort, 9% in a Thai cohort, 78% in an Indian cohort, and 55% in our mostly black African cohort [14, 15, 18]. Mild and moderate pigmentation likely represents complexion associated melanosis. With severe pigmentation, the differential diagnosis should include a naevus, melanoma and OSSN. Conjunctival melanoma is an uncommon presentation in the black South African population, and so a high index of suspicion should be maintained for OSSN [19]. Morphology was a further feature distinguishing OSSN from benign lesions. A fibrovascular lesion was more likely to be benign, whereas a leukoplakic or gelatinous lesion was more likely to be OSSN. Kaliki et al. [14] in India and Tananuvat et al. [15] in Thailand found papilliform lesions to be the most common morphological type, whereas Gichuhi et al. [17] in Kenya found a predominance of leukoplakic lesions. This regional difference may be attributed to different associated risk factors such as HPV infection, which could be reviewed in further studies.

The histology profile of OSSN has significant regional differences. Low-income countries have a predominance of SCC whereas high-income countries have a predominance of CIN (Table 4). Our study had a predominance of CIN lesions, which differs from other African countries with a predominance of SCC [20–22]. Our study was conducted in an urban environment where there is easy access to healthcare services. This is reflected by the short delay of 2 days between presentation to a primary care centre and referral to our ophthalmic unit. This could explain the predominance of earlier histological grades of OSSN in our cohort. OSSN has been described as an incidental finding in pterygium surgery in 10% of cases [23]. Our study found OSSN in 31% of cases that were clinically suspected to be pterygia. This highlights the importance of sending all specimens for histology to rule out OSSN.

HIV has been reported as an important association with OSSN, notably in the African region where prevalence rates are high [8, 17, 21, 24–26]. The overall prevalence of HIV in our study was 61%. The cases had a prevalence of 74% while the controls had a prevalence of 32%. This is in keeping with other studies in Africa where prevalence rates of HIV in OSSN ranged from 74–79% [8, 12, 17, 24]. In HIC this is an uncommon association with prevalence rates of 3% in the United States [27]. Although this highlights the importance of HIV as an associated risk for developing OSSN, this also means that 26% of OSSN patients in our group did not have HIV as a risk factor. There was a significant association between a lower CD4 and higher viral load for OSSN, but this did not hold up with regression analysis. There was a decreasing trend of CD4 count with more advanced histology of OSSN. Gichuhi et al. [8] also described an association between OSSN and CD4, with an OR of 37 when comparing a CD4 of <200 and >500, albeit it with a large confidence interval (95% CI: 7.98–174.5).

Other associated factors reviewed in our study included reported sun exposure, ocular trauma, history of ocular inflammatory disease, exposure to petroleum products, vitamin A levels and hepatitis infections. These were not shown to be statistically different between cases and controls. Of these, sun exposure is a known risk factor for the development of pterygia which made up most of the controls [28]. It would therefore be difficult to draw any conclusions on the role of sun exposure as a risk factor for the cases in our group. South Africa is situated at the edge of the 30-degree high risk UV belt at 28.2 degrees, so it may be that sun exposure is not a significant risk factor in our cohort. The other risk factors we evaluated are not known associations with pterygia and therefore are also not likely associations with OSSN in our study population [28]. This brings to the fore the importance of other associated factors such as HPV infection.

There are several limitations in this study. Recruitment occurred predominantly during the course of the COVID19 pandemic and was consequently affected by waves of infection where all research activity was halted. Cases were therefore not recruited from consecutive cases that presented to the clinic. We do not think that this would have resulted in selection bias, as many patients did not present during the peak waves of infection and there was consecutive recruitment in between waves. Several components of the questionnaire were subject to recall bias, such as duration of symptoms, when the mass was first noticed, diagnosis and initiation of treatment for HIV, previous ocular history, tobacco use, and sun exposure. Sun exposure only took into account the average amount of time in the sun over the recent months. This therefore did not account for previous sun exposure in younger years. Tumour thickness was compared to the eyelid as UBM was not available during the study. This is a crude method of measurement and used as a surrogate for UBM. This is not expected to have any effect of our results. In the HIV positive patients with a known diagnosis, data were not available from the initial point of diagnosis. This would have been a useful metric to determine if the nadir CD4 and viral loads had any impact on disease presentation. The controls only had 14 patients with HIV. This limited a more in-depth analysis of the effects of CD4, viral load and the effect of anti-retro-viral therapy. This was however a large group of patients with OSSN and represents the largest study conducted in South Africa.

Our study describes an OSSN cohort of a MIC, but with a lower age due to the high prevalence of HIV. The majority of cases presented with CIN lesions, rather than SCC reported in other African countries. Severe pigmentation was present more commonly than groups with a predominantly white patient profile. In black patients OSSN should be considered before melanoma when presenting with a clinically suspicious pigmented conjunctival mass. These regional differences are important to consider when diagnosing OSSN. In keeping with other studies in Africa, HIV was the leading risk factor. Future studies could investigate the role of CD4 and viral load in an HIV cohort and the role of HPV.

## Summary

### What was known before

- Ocular surface squamous neoplasia (OSSN) is the most common ocular surface tumour.
- The two main demographic groups have classically been described are older white males in developed countries and younger black females in developing countries.
- Ultraviolet-B and HIV are the leading risk factors.

### What this study adds

- There is a distinct difference in demographic characteristics of OSSN patients in high-, middle- and low-income countries. It is therefore necessary to update the classification from the traditional developed and developing stratification into these three groups.
- High-income countries have a predominantly older white male OSSN demographic.
- Middle-income countries have a younger OSSN demographic with no gender predisposition.
- Low-income countries have a younger predominantly female cohort of OSSN patients.
- South Africa falls into the middle-income group, but with a lower median age, due to the high prevalence of HIV.

### How this study might affect research, practice or policy

- Future studies could:
  - Further investigate the relationship between income, risk factors and OSSN.
  - Investigate the role of CD4 and viral load in an HIV cohort.
- The large role that HIV plays in OSSN is an important consideration for a country’s anti-retroviral therapy programme.

### Supplementary information

Supplement 1

## Data Availability

Data are available from the corresponding author on request.
