# Supplementary material for: Demographics, clinical presentation and risk factors of ocular surface squamous neoplasia at a tertiary hospital, South Africa
Source: Eye (Lond). 2023 May 31;37(17):3602–8. doi: 10.1038/s41433-023-02565-1 (PMC10686408; doi:10.1038/s41433-023-02565-1)
Supplement: Supplementary file 1 — Supplement 1 [file 41433_2023_2565_MOESM1_ESM.docx]

## **Supplements**

Assessed for eligibility (n=191)

Included in the study (n=175)

Excluded (n=16)

Not meeting inclusion criteria (n=7)

Declined to participate (n=3)

Other reasons (n=6)

Controls (n= 45)

Cases (n= 130)

Biopsy (n= 175)

Supplement 1: Flow diagram of recruitment and study participants
